# Identification of a novel mutation in *PEX10* in a patient with attenuated Zellweger spectrum disorder: a case report

**DOI:** 10.1186/s13256-017-1365-5

**Published:** 2017-08-08

**Authors:** Maria Blomqvist, Karin Ahlberg, Julia Lindgren, Sacha Ferdinandusse, Jorge Asin-Cayuela

**Affiliations:** 1 Institute of Biomedicine, Department of Clinical Chemistry and Transfusion Medicine, Sahlgrenska Academy, Gothenburg University, Gothenburg, Sweden; 2 Paediatric Clinic, Central Hospital, S-65185 Karlstad, Sweden; 3 Laboratory Genetic Metabolic Diseases, Department of Clinical Chemistry, Academic Medical Center, Amsterdam, The Netherlands

**Keywords:** Zellweger spectrum disorder, Peroxisome biogenesis disorder, PEX10, Case report

## Abstract

**Background:**

The peroxisome biogenesis disorders, which are caused by mutations in any of 13 different *PEX* genes, include the Zellweger spectrum disorders. Severe defects in one of these *PEX* genes result in the absence of functional peroxisomes which is seen in classical Zellweger syndrome. These patients present with hypotonia and seizures shortly after birth. Other typical symptoms are dysmorphic features, liver disease, retinal degeneration, sensorineural deafness, polycystic kidneys, and the patient does not reach any developmental milestones.

**Case presentation:**

We report a case of a patient with Zellweger spectrum disorder due to a novel mutation in the *PEX10* gene, presenting with a mild late-onset neurological phenotype. The patient, an Assyrian girl originating from Iraq, presented with sensorineural hearing impairment at the age of 5 followed by sensorimotor polyneuropathy, cognitive delay, impaired gross and fine motor skills, and tremor and muscle weakness in her teens. Analyses of biochemical markers for peroxisomal disease suggested a mild peroxisomal defect and functional studies in fibroblasts confirmed the existence of a peroxisome biogenesis disorder. Diagnosis was confirmed by next generation sequencing analysis, which showed a novel homozygous mutation (c.530 T > G (p.Leu177Arg) (NM_153818.1)) in the *PEX10* gene predicted to be pathogenic.

**Conclusions:**

This case highlights the importance of performing biochemical, functional, and genetic peroxisomal screening in patients with clinical presentations milder than those usually observed in Zellweger spectrum disorders.

## Background

Peroxisomes are subcellular organelles present in all eukaryotic cells except for mature erythrocytes. A range of different metabolic functions are specific for this organelle, that is, beta-oxidation of very long chain fatty acids (VLCFA) and pristanic acid, alpha-oxidation of phytanic acid, biosynthesis of etherphospholipids, bile acids and docosahexaenoic acid, and glyoxylate detoxification [1]. The importance of peroxisomes in metabolism is evident by the existence of a large group of genetic diseases associated with impaired peroxisome biogenesis and function [2]. Peroxisomal disorders are divided into two groups: (1) peroxisome biogenesis disorders (PBDs), and (2) single peroxisomal enzyme defects. PBDs include the Zellweger spectrum disorders (ZSDs) and rhizomelic chondrodysplasia punctata type 1 and 5. ZSDs have autosomal recessive inheritance and are caused by mutations in any of 13 different *PEX* genes [3]. These *PEX* genes encode for proteins called peroxins that are involved in peroxisome biogenesis and/or protein import [4]. Severe defects in one of these *PEX* genes result in the absence of functional peroxisomes which is seen in classical Zellweger syndrome, while milder phenotypes are often associated with partial loss of peroxisomal function.

Patients with ZSD show neurodevelopmental disease that ranges from pronounced neuronal migration deficiencies with hypotonia, seizures, dysmorphic features, liver disease, retinal degeneration, sensorineural deafness, and polycystic kidneys to a more attenuated phenotype [2, 3]. Patients with the classical presentation show elevated plasma levels of VLCFAs, pipecolic acid and C_27_ bile acid intermediates together with diminished erythrocyte etherphospholipids (plasmalogens). Phytanic and pristanic acid levels are normal at birth, but can be elevated depending on dietary intake. Cultured fibroblasts from these patients show increased levels of VLCFAs, deficient plasmalogen biosynthesis, and C26:0, phytanic and pristanic acid oxidation. Cytochemical evaluation shows mislocation of the peroxisomal matrix protein catalase to the cytosol. Patients with a milder clinical presentation often show less pronounced abnormalities of peroxisomal metabolites in body fluids and fibroblasts [5, 6]. Among ZSD patients *PEX1* is the most affected gene, followed by *PEX 6* and *PEX12* [7]. This report describes a new missense mutation in *PEX10* in a patient with mild clinical phenotype and highlights the importance of performing biochemical and genetic peroxisomal screening in patients with clinical presentations milder than those observed in ZSDs.

## Case presentation

**Fig. 1:**
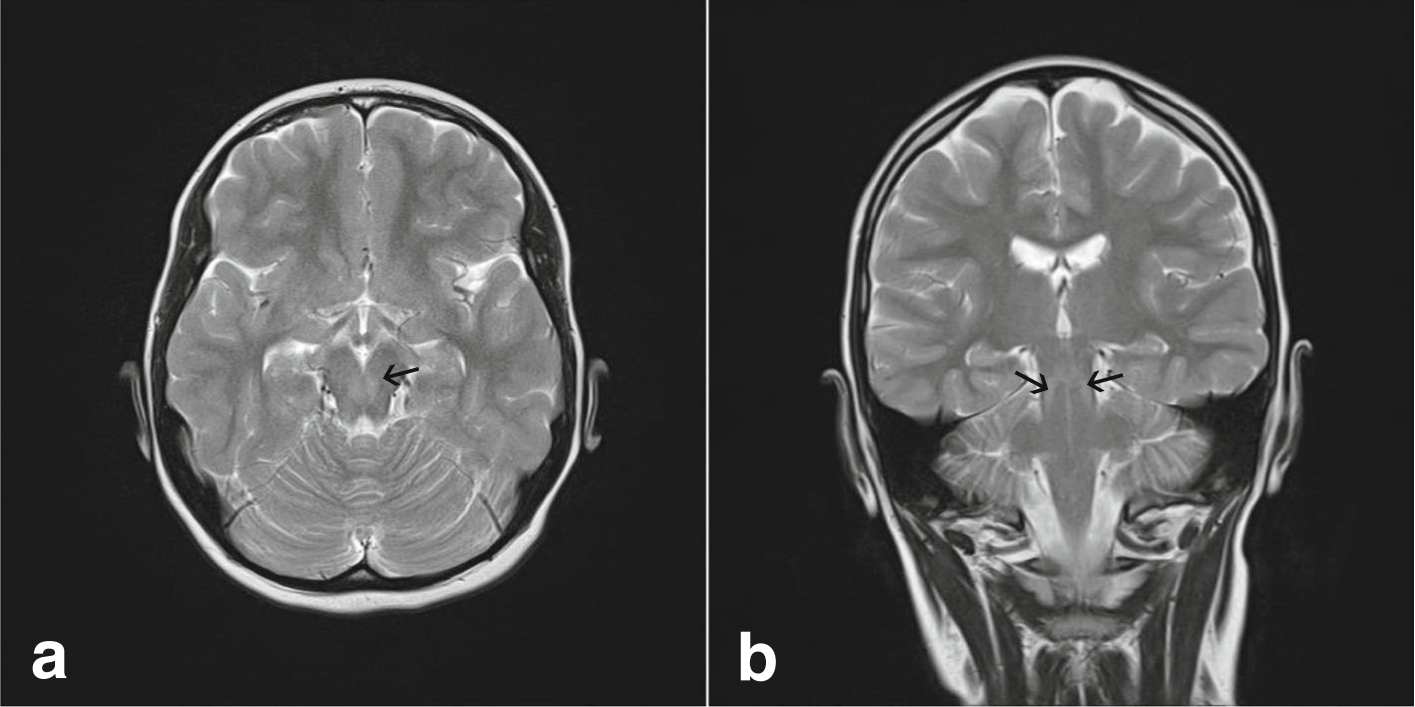
Magnetic resonance imaging scans from patient with PEX10 defect (12 years of age). The magnetic resonance imaging scan shows: **a** axial T2-weighted imaging and **b** coronal T2-weighted imaging revealing a central lesion in the mesencephalon (*arrows*), from the nucleus ruber dorsocaudally down to the cerebellar peduncles

Our patient is a 15-year-old girl born in Sweden after 40 weeks gestation as the third of four siblings from Assyrian consanguineous parents originating from Iraq. Contact with the national health system was sparse until the age of 5 years, when she presented with sensorineural hearing impairment and developmental delay. After application of bilateral hearing aids her development accelerated in all domains. In retrospect her parents reported frequent loss of balance as a toddler. Our patient was referred from her school physician to a child neurologist at the age of 7 years and 8 months due to her toe-walking gait. Except for her Achilles tendons being tight, almost spastic, no other neurological abnormality was noted. Brain imaging at the age of 8 years showed a central lesion in the mesencephalon, from the nucleus ruber dorsocaudally down to the cerebellar peduncles. A magnetic resonance imaging (MRI) scan of her spine was normal. After physiotherapy and orthoses her gait improved, but her motor skills were still abnormal for her age. At the age of 12 years she showed bilateral muscle weakness on her peroneal muscles with absence of peripheral reflexes. Neurography and electromyography showed sensorimotor polyneuropathy and chronic neurogenic changes in her leg muscles. An MRI scan of the brain was repeated showing the same lesion as described at the age of 8 years (Fig. 1). At the age of 13 she presented cognitive delay, impaired gross and fine motor skills, and tremor. At this point, a neurometabolic disease was considered. At the age of 14 she presented learning difficulties and obvious generalized muscle weakness. Her fingers are extremely flexible, her feet are in plan valgus position and she has problems with her balance. At the age of 15 she shows no signs of puberty. Growth is also delayed with a skeletal age of 10.7 at chronological age of 14. Hormonal tests have shown nonfunctioning ovaries. She has no retinal changes and normal optic nerves. One of the younger sisters presents some learning difficulties.

Blood metabolites were analyzed twice when our patient was 13 years (Table 1). Her plasma levels of C26:0 were moderately increased resulting in an increased C26:0/C22:0 ratio. The C24:0/C22:0 ratio was borderline normal. Her phytanic and pristanic acid levels were significantly increased at both sampling occasions. Her erythrocyte plasmalogen levels were normal. Because of the abnormal VLCFA and phytanic and pristanic acid levels further peroxisomal studies in fibroblasts were performed. Remarkably, her VLCFAs and DHAPAT activity in cultured fibroblasts were normal (Table 2).

**Table 1:** Blood analytes

Plasma analyte	Sampling 1	Sampling 2	Normal range
C26:0 (μmol/l)	1.78	2.19	0.3–1.0
C26:0/C22:0	0.046	0.061	0.006–0.021
C24:0/C22:0	0.89	1.01	0.49–0.91
Phytanic acid (μmol/l)	26.2	40.1	<10
Pristanic acid (μmol/l)	67.2	74.1	<1.5
C16DMA/C16:0	0.08	-	0.02–0.06
C18DMA/C18:0	0.17	-	0.06–0.18

**Table 2:** Fibroblast studies

Biomarker fibroblasts	Patient	Normal range
C22:0 (μmol/g protein)	3.79	2.46–6.59
C24:0 (μmol/g protein)	8.16	6.37–13.87
C26:0 (μmol/g protein)	0.22	0.16–0.41
C24:0/C22:0	2.15	1.68–2.92
C26:0/C22:0	0.06	0.03–0.10
Dihydroxyacetonephosphate-acyltransferase activity (DHAPAT) (nmol/(2 hour.mg protein)	10.6	5.9–15.5

**Fig. 2:**
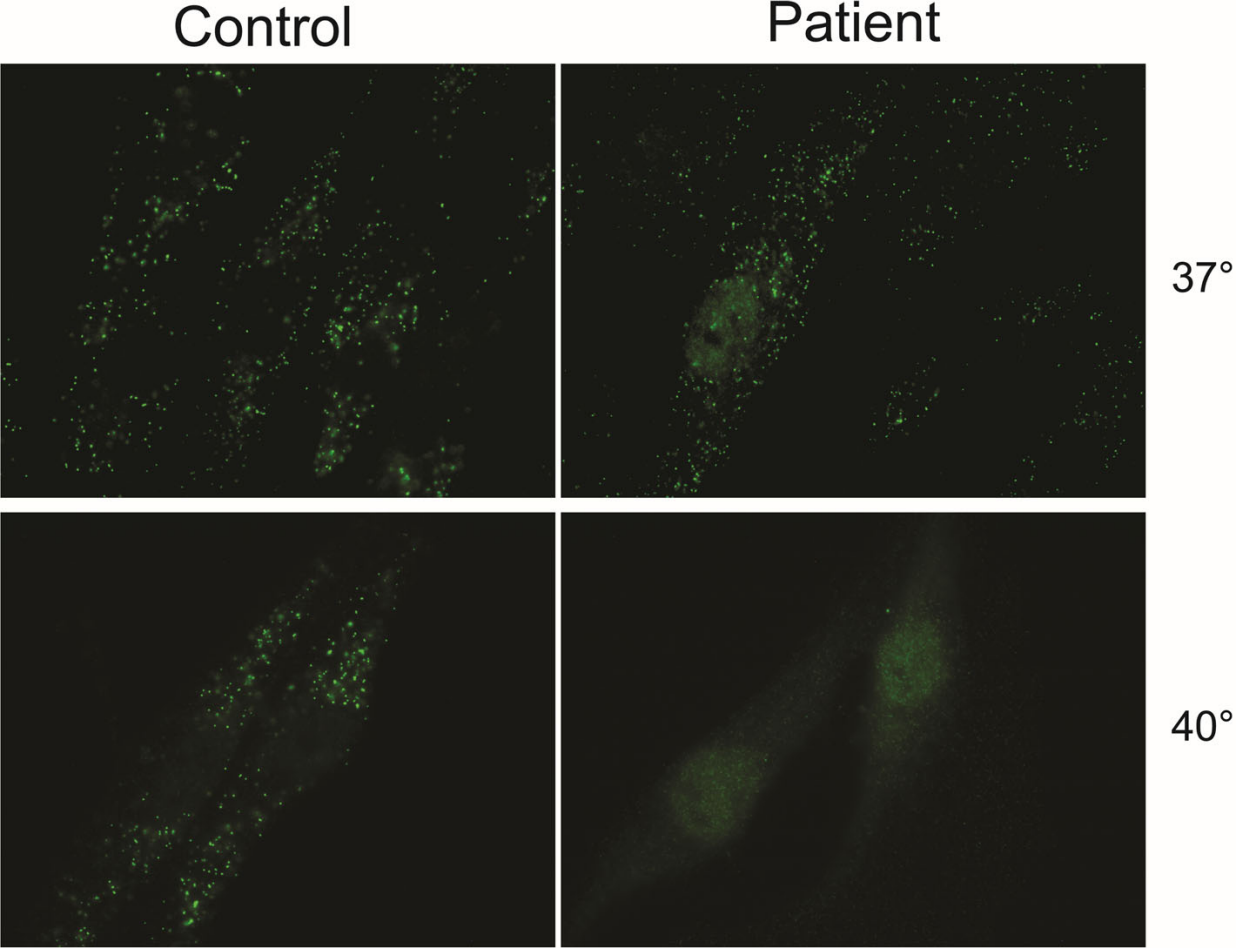
Immunofluorescence microscopy analysis using antibodies raised against catalase, a peroxisomal matrix enzyme, in skin fibroblasts of a control subject (*left panels*) and the patient, F1453 (*right panels*) cultured at 37 °C (*upper panels*) and 40 °C (*lower panels*) for 2 weeks. At 37 °C cells of the patient reveal a normal peroxisomal staining in most cells (a representative picture is shown), whereas at 40 °C catalase staining becomes cytosolic confirming the peroxisome biogenesis defect in the patient

Immunofluorescence microscopy analysis using antibodies raised against catalase, a peroxisomal matrix protein, did show abnormal peroxisomal staining but not in all cells. When cultured at 37 °C most cells revealed a normal peroxisomal staining, but in some cells a markedly reduced number of peroxisomes was observed. Culturing patient fibroblasts at 40 °C for 2 weeks resulted in a complete absence of peroxisomal staining with catalase immunofluorescence microscopy analysis, indicating the lack of import-competent peroxisomes at this elevated temperature (Fig. 2). These results showed that our patient suffered from a Zellweger spectrum defect.

**Fig. 3:**
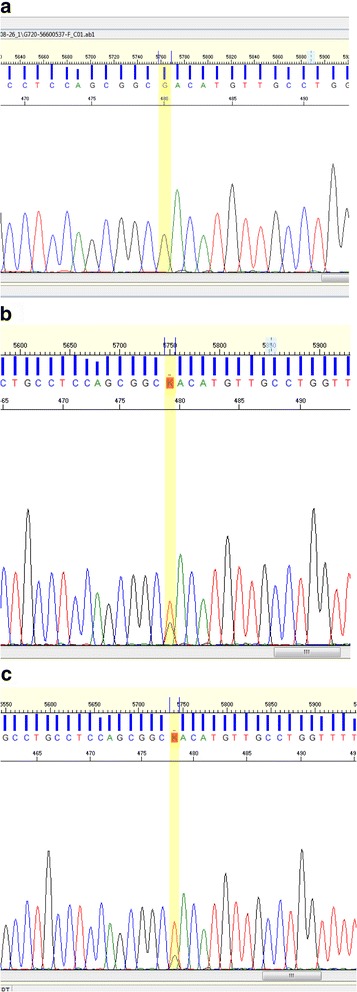
Sequencing chromatograms confirming the next generation sequencing findings and showing expected segregation pattern. **a** Patient, homozygous for c.530 T > G (p.Leu177Arg) in *PEX10* gene. **b** and **c** Father and mother, heterozygous for the same variant

Mutation analysis was performed by using a custom-made next generation sequencing (NGS) gene panel (SureSelect^QXT^, Agilent Technologies, Santa Clara, CA, USA). The panel targets coding exons of 21 genes described to be involved in peroxisomal disorders including the 13 *PEX* genes (+/- 25 bases, according to RefSeq database and assembly Feb. 2009 (GRCh37/hg19)). Variants of interest were filtered according to allele frequency, exonic/splice site location and autosomal recessive or X-linked pattern of inheritance. NGS analysis identified only one variant of potential clinical significance, namely a homozygous mutation (c.530 T > G (p.Leu177Arg) (NM_153818.1)) in the *PEX10* gene. This variant is not present in HGMD® Professional database (Biobase, Qiagen®, Redwood City, CA, USA) or ClinVar [8], but has a very low allele frequency in the normal population according to 1000 genome database [9], is classified as probably damaging by PolyPhen and deleterious by SIFT and affects a highly conserved amino acid. The result was verified by Sanger sequencing and targeted sequencing on the parents showed the expected segregation pattern (Fig. 3). To confirm the PEX10 defect in the patient we performed complementation studies at 40 °C by co-transfection of wild-type PEX10 and PTS1 (peroxisome targeting sequence 1)-tagged green fluorescent protein (eGFP-SKL) in the patient’s fibroblasts. Three days after transfection there was restoration of PTS-1 protein import, confirming that PEX10 is responsible for the peroxisomal dysfunction in our patient (data not shown).

## Discussion

The ZSDs fall into three main phenotypic classes, the Zellweger syndrome, neonatal adrenoleukodystrophy and infantile Refsum disease (IRD), with the most severe being the Zellweger syndrome and the least severe IRD. However, even milder clinical forms of non-IRD ZDS have been reported, for example in patients with *PEX1* or *PEX6* defects [10–12]. Most of these patients presented biochemical profiles in blood and cultured fibroblasts characteristic for ZSD, but some did not show the complete characteristic biochemical profile. In this report, we describe a patient with *PEX10* deficiency showing non-IRD mild clinical phenotype together with an incomplete biochemical profile. This further highlights the importance of molecular analysis for the diagnosis of these patients [13–16].

So far, about 30 pathogenic variants have been reported in *PEX10* [7]. Mutations are distributed throughout the entire gene, with exon 5 harboring most mutations. The majority of the reported patients with mutations in *PEX10* belong to the severe Zellweger syndrome or neonatal adrenoleukodystrophy [17], but also milder clinical forms with mutations in *PEX10* have been described. Five patients described with mild clinical presentation caused by *PEX10* variants all show cerebellar ataxia [18, 19], suggesting PBD to be considered in the differential diagnosis of autosomal recessive ataxia. Genotype-phenotype correlation has been suggested for PEX10 deficiency, where nonsense and frameshift mutations seem to be associated with severe clinical and cellular phenotypes while missense mutations are associated with milder phenotypes [14, 18, 20, 21]. The case we present in this report supports this hypothesis.

Peroxisomal mosaicism, that is, a mixed population of fibroblasts with normal peroxisome numbers and reduced numbers of peroxisomes, was observed in our patient. This phenomenon has previously been described in other patients with mild mutations in *PEX10* [14, 18] as well as other *PEX* genes. Culturing fibroblasts at 40 °C usually aggravates the defect, an effect that was also found in our study where a complete absence of peroxisomal staining with catalase immunofluorescence microscopy analysis was observed. This result showed that there is a lack of import-competent peroxisomes at this elevated temperature. Overall, the results observed in fibroblasts suggest that the mutated PEX10 protein can partly be correctly targeted to the peroxisomal membrane and has residual activity, which is in line with the biochemical and clinical presentation of our patient.

Proteins synthesized in the cytosol containing the PTS1-tag form a complex with PEX5 prior to import into the peroxisome. After import of the cargo protein, PEX5 is either mono-ubiquitinated and recycled back to the cytosol or poly-ubiquitinated and degraded by the proteasome. PEX10, which has ubiquitin ligase activity, is required, together with PEX2 and PEX12, for the ubiquitination of PEX5 in the peroxisomal membrane [22]. PEX10 contains a conserved zinc finger domain located at the C-terminus called RING finger. This domain is shared by many ubiquitin ligases and has been shown to be essential for the ubiquitin ligase activity of PEX10 [23]. The majority of described variants of PEX10 defects are located in the coding region of this specific domain. Furthermore, other RING ligases require additional domains, like G2BR [24] or CUE, a three-helix bundle that binds ubiquitin via conserved hydrophobic residues [25]. The mutation reported here substitutes the leucine at position 177 (L177) for an arginine and is not located in the RING finger domain. Since only the RING finger domain has been crystalized, we can only speculate as to the possible structural effect of this substitution. L177 is located in a highly hydrophobic stretch predicted to be a transmembrane domain by several prediction softwares [26, 27]. In that case, the substitution of a hydrophobic leucine for a positively charged arginine could have important consequences for the interaction of PEX10 with the peroxisomal membrane. Alternatively, since both G2BR and CUE domains are rich in hydrophobic residues, it is tempting to suggest that L177 might be part of a similar domain in PEX10, in which case a mutation in that position could have significant functional consequences.

## Conclusions

We describe a patient affected by a ZSD with a mild clinical and biochemical phenotype caused by a novel homozygous mutation in the *PEX10* gene (c.530 T > G (p.Leu177Arg) (NM_153818.1)). This case report highlights the importance of performing biochemical and genetic peroxisomal screening in patients with clinical presentations milder than those usually observed in ZSDs.

## Abbreviations

IRD: Infantile Refsum disease
MRI: Magnetic resonance imaging
NGS: Next generation sequencing
PBD: Peroxisomal biogenesis disorders
PTS1: peroxisome targeting sequence 1
VLCFA: Very long chain fatty acids
ZSD: Zellweger spectrum disorders
